# RRBP1 overexpression is associated with progression and prognosis in endometrial endometrioid adenocarcinoma

**DOI:** 10.1186/s13000-019-0784-6

**Published:** 2019-01-26

**Authors:** Shuang Liu, Mu Lin, Hongying Ji, Jing Ding, Jiaqi Zhu, Rong Ma, Fanling Meng

**Affiliations:** Department of Gynecology, Harbin Medical University Cancer Hospital, Harbin, 150081 China

**Keywords:** Ribosome-binding protein 1 (RRBP1), Endometrial cancer (EC), Diagnosis, Prognosis

## Abstract

**Background:**

Currently, ribosome-binding protein 1 (RRBP1) is considered to be a novel oncogene that is overexpressed in colorectal cancer, lung cancer, mammary cancer, esophageal cancer and other carcinomas. However, the relationship between RRBP1 and endometrioid-type endometrial carcinoma (EC) remains unknown. Our purpose is to explore the function of RRBP1 in endometrioid-type endometrial carcinoma.

**Methods:**

We investigated the expression of RRBP1 protein by immunohistochemistry on paraffin-embedded surgical specimens from one hundred thirty patients with endometrioid-type endometrial carcinoma. We also evaluated the differences in RRBP1 expression between endometrial cancer samples (*n* = 35) and normal endometrial tissues (*n* = 19) by western blotting.

**Results:**

RRBP1 was more highly expressed in endometrial cancer samples than in normal samples (*P* < 0.05). High levels of expression of RRBP1 were strongly correlated with pathological features, such as the Federation of Gynecology and Obstetrics (FIGO) stage, histological grade, depth of myometrial invasion and lymph node metastasis (*P* < 0.05). Furthermore, RRBP1 expression was an independent prognostic factor for overall survival (OS) and disease-free survival (DFS) in patients with EC (both P < 0.05).

**Conclusion:**

This experiment identifies the utility of RRBP1 in predicting EC prognosis, revealing that it may be a potential target for therapeutics of EC.

## Background

Endometrial cancer (EC) is the most common gynecological cancer in developed countries [1]. Recent data published by the American Cancer Society reported that 63,230 new cases are expected to occur in 2018, with an estimated 11,350 women dying from the disease [2]. If endometrial cancer can be diagnosed early, it can be treated surgically alone or in combination with adjuvant chemotherapy or radiation [3]. However, it is still a significant clinical challenge to treat advanced and recurrent patients [4–6]. It would be very helpful if medical researchers could find prognostic molecular biomarkers for advanced and recurrent endometrial cancer.

RRBP1 is an endoplasmic reticulum membrane protein that is critical for the transportation and secretion of nascent proteins in mammalian cells [7]. Recently, RRBP1 has been confirmed to be overexpressed in lung cancer [8], breast cancer [9], colorectal cancer [10], and esophageal cancer [11]. RRBP1 overexpression promotes the progression of esophageal cancer and colorectal cancer, and is helpful for predicting patient outcomes. In Her-2-positive patients, high RRBP1 expression is correlated with a poor overall survival, and it can be an independent predictor of survival [12]. However, the expression and clinical significance of RRBP1 in endometrial carcinoma has not previously been reported.

This study aimed to determine RRBP1 expression in endometrioid-type endometrial carcinoma and to reveal the connection between RRBP1 and the clinical significance of endometrial cancer.

## Methods

### Study population

Our research was approved by the Ethical Committee of the Harbin Medical University Cancer Hospital,and informed consent for tissue experiment was obtained from all patients.In this study, we examined one hundred thirty endometrial endometrioid adenocarcinoma patients from January 2010 to May 2013 who were treated at Harbin Medical University Cancer Hospital and underwent hysterectomy, bilateral salpingo-oophorectomy, pelvic and/or paraaortic lymphadenectomy, partial omentectomy, and peritoneal washing for cytology. None of them had received chemotherapy or radiation before the surgery. We assessed the stage of their endometrial cancer based on the International Federation of Obstetricians and Gynecologists (FIGO) [13] guidelines and assessed the histological grade in light of the WHO histopathological grading system standards [14]. Normal tissues in this research were selected from among people who underwent hysterectomy for hysteromyoma at the Department of Gynecology of the Harbin Medical University Cancer Hospital. Fresh tissues from 35 patients, including tumor tissues (*n* = 35) and normal tissues (*n* = 19), were collected and stored at − 80 °C for the western blot experiment.

### Follow up information

All of the endometrial cancer patients were followed up for survival analysis until December 1, 2016. They were followed-up for a mean of 58.94 months (range, 35–83 months).

### Western blot analysis

Fifty four samples were frozen and then homogenized in RIPA buffer (Abcam, Cambridge, MA, USA), and the desired protein samples were collected. The appropriate amount of sample buffer was added to the collected protein samples and heated at 100 °C in a boiling water bath for 3–5 min to fully denature the protein. After cooling to room temperature, the protein sample was electrophoretically separated on a 10% sodium dodecyl sulfate polyacrylamide gel and then the proteins were transferred to a polypropylene fluoride membrane (Millipore, Billerica, MA, USA). After the completion of the transfer of the membrane, it was immediately placed in a previously prepared Western washing solution and rinsed for 1–2 min to wash the transferring solution off the membrane. The antibodies used in this study include anti-RRBP1 antibody (1:300, Abcam, Cambridge, MA, USA) and anti-β-actin antibody (1:1000, Santa Cruz Biotechnology, Santa Cruz, CA, USA).

### Immunohistochemical staining and assessment

The embedded samples were cut into 4-μm sections and stained with hematoxylin. After dewaxing in xylene, the slides were dehydrated. The sections in 0.3% hydrogen peroxide were incubated in the dark for 10 min at room temperature to block endogenous peroxidase activity and then conducted antigen repair in 6 mmol/L sodium citrate buffer (pH 6.0) (Mitsubishi Chemical Medical Corporation, Tokyo, Japan) at a temperature higher than 100 °C for 4 min. After washing with Phosphate-Buffered Saline(PBS) the slides were wiped dry. Then, they were placed in a humid chamber and incubated with blocking solution (BSA) for 20 min at room temperature. The blocking solution was blotted off and the sections were incubated with a 1:100 dilution of anti-RRBP1 antibody (1:300, Abcam, Cambridge, MA, USA) overnight at 4 °C. The slides were washed with PBS, wiped and placed in a humid chamber and then incubated at room temperature with the secondary antibody for 20 min. The slide was washed again with PBS, wiped, and placed on a wooden board. Diaminobenzidine (DAB) staining was then performed.

The staining was observed under a microscope, and then the staining was stopped by immersing it in PBS. Hematoxylin counterstaining was applied. According to the number of positive tumor cells, the staining was scored as follows: ‘0’ < 5%, ‘1’ 5–24%, ‘2’ 25–49%, and ‘3’ 50–100%. The intensity of the staining was scored as blank (0), weak (1), moderate (2), and strong (3). Based on the percentage of positively stained tumor cells and the staining intensity, a semiquantitative classification of RRBP1 protein expression levels was scored as < 4 indicates low expression and ≥ 4 indicates high expression [11].

We invited two independent pathologists to calculate the immunohistochemistry scores in duplicate for each slide. They were experienced pathologists who were good at assessing immunohistochemistry and were blinded to any clinicopathological information about the slides.

### Statistical analysis

Chi-square tests were used to analyze the relationship between RRBP1 and the clinicopathological parameters. The Kaplan–Meier method and log-rank test were used for survival analysis. Cox proportional hazards regression was performed for the multivariate analysis of prognostic factors.

## Results

### RRBP1 was overexpressed in EC

**Fig. 1:**
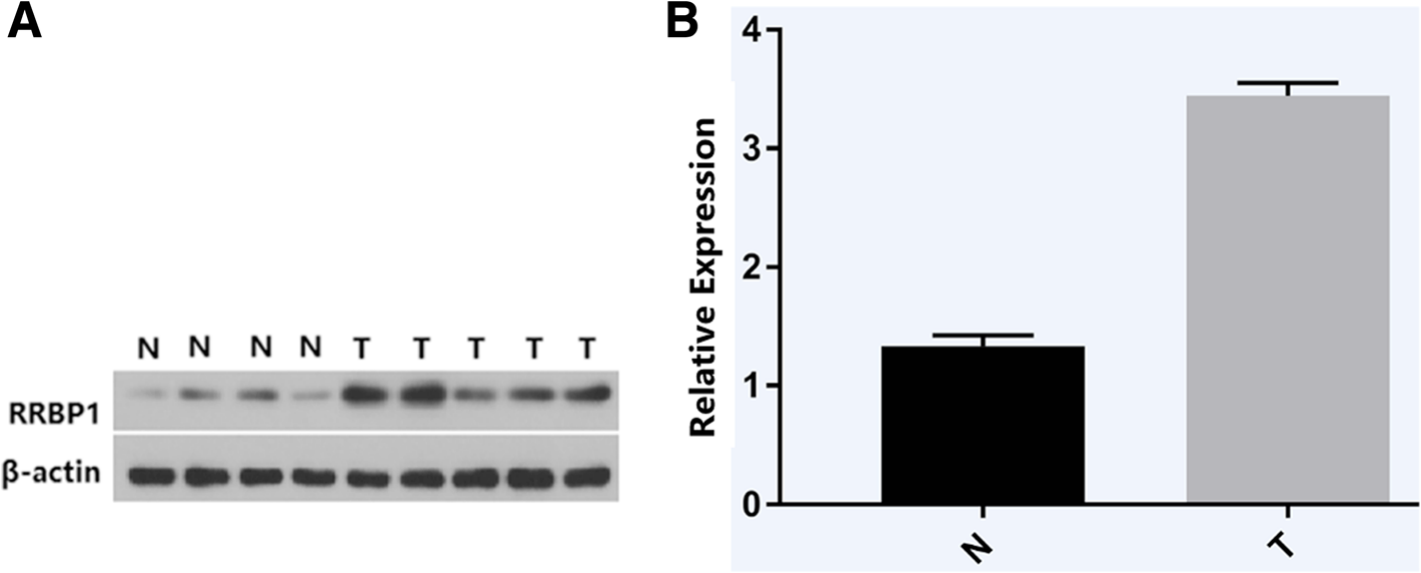
**a**, Representative protein samples obtained from frozen normal endometrial tissues (N) and endometrial endometrioid adenocarcinoma tissues (T) were analyzed by Western blot analysis. The levels of β-actin were used as an internal control; **b**, Histogram of pooled data from N (*n* = 19) and ECs (*n* = 35). RRBP1 expression was elevated in ECs compared with N. The data are presented as mean ± s. d. (*P* < 0.05)

Western blot analysis showed that RRBP1 was highly expressed in EC tissues and weakly expressed in normal tissues (*P* < 0.05, Fig. 1).

**Fig. 2:**
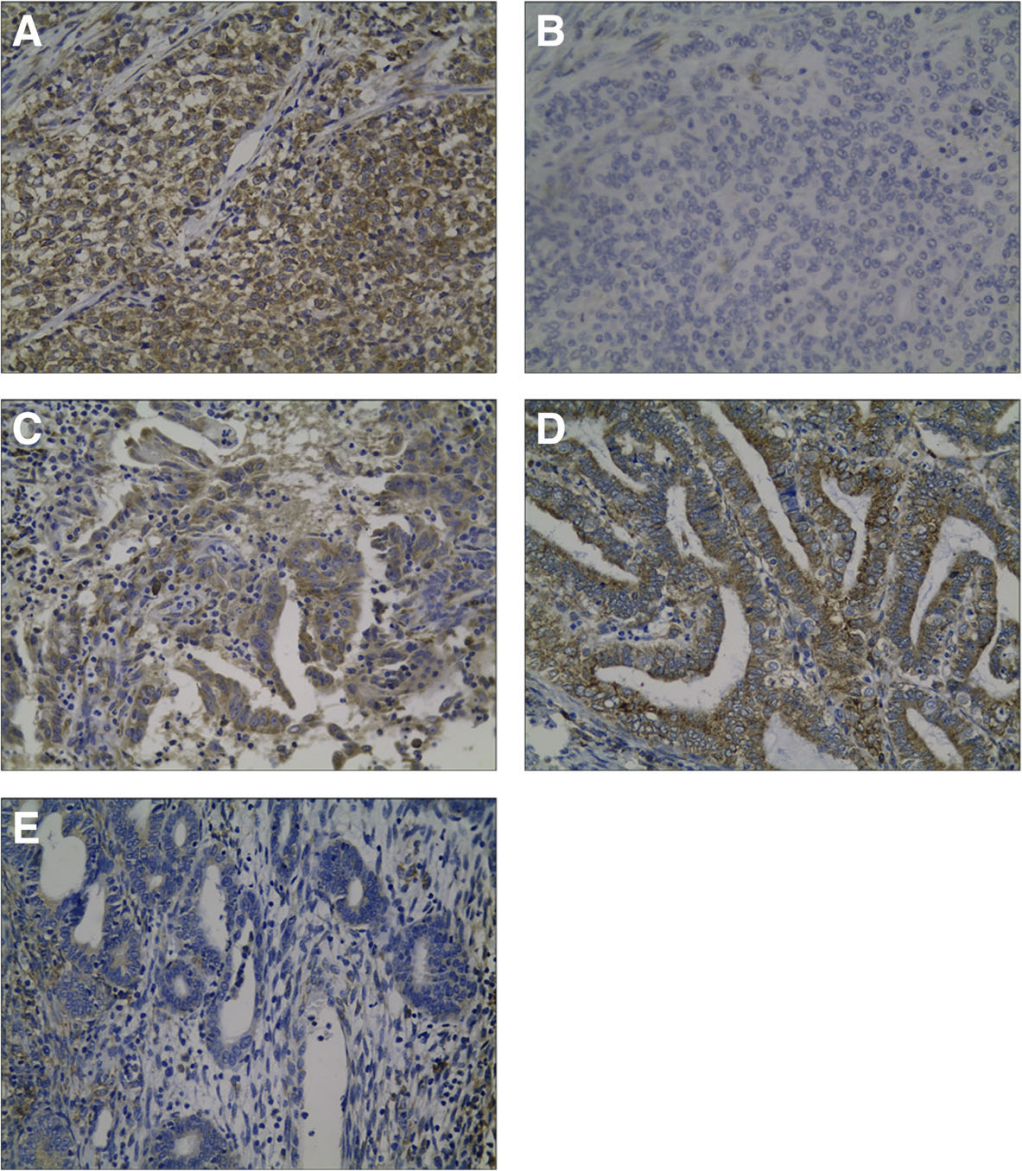
Representative immunohistochemical staining of RRBP1 in endometrial endometrioid adenocarcinoma specimens (EC): **a**, Low expression of RRBP1 in a poorly differentiated EC; **b**, High expression of RRBP1 in a poorly differentiated EC; **c**, High expression of RRBP1 in a moderatedly differentiated EC; **d**, High expression of RRBP1 in a well differentiated EC; **e**, Low expression of RRBP1 in a well differentiated EC

Immunohistochemical analysis showed expression of RRBP1 in the cytoplasm of EC tissues (Fig. 2). High expression of RRBP1 in EC tissues was obviously correlated with high FIGO stages (*P* = 0.003), deep muscular layer infiltration (*P* < 0.001), and high histological grades (*P* = 0.005) (Table 1). RRBP1 expression was also significantly increased in EC patients with lymph node metastasis (*P* = 0.021) (Table 1).

**Table 1:** Association analyses between the expression levels of RRBP1 and the clinicopathological characteristics of endometrial endometrioid adenovarcinoma

Variables	Patients *n*	RRBP1 expression		*P* ^a^
		Low	High	
All cases				
Age(years)				
<60	93	37	56	*P* = 0.432
≥ 60	37	18	19	
FIGO stage				
I	103	51	52	*P* = 0.003
II	17	4	13	
III	10	0	10	
Histological grade				
G1	42	25	17	*P* = 0.005
G2~G3	88	30	58	
Lymph node metastasis				
No	122	55	67	*P* = 0.021
Yes	8	0	8	
Deep muscular layer infiltration				
<50%	107	54	53	*P*<0.001
≥ 50%	23	1	22	

FIGO, International Federation of Gynecology and Obstetrics; G1, well differentiated; G2, moderately differentiated; G3, poorly differentiated; RRBP1, Ribosome-binding protein 1; ^a^Chi-square test

### Prognostic significance of RRBP1 in EC

**Fig. 3:**
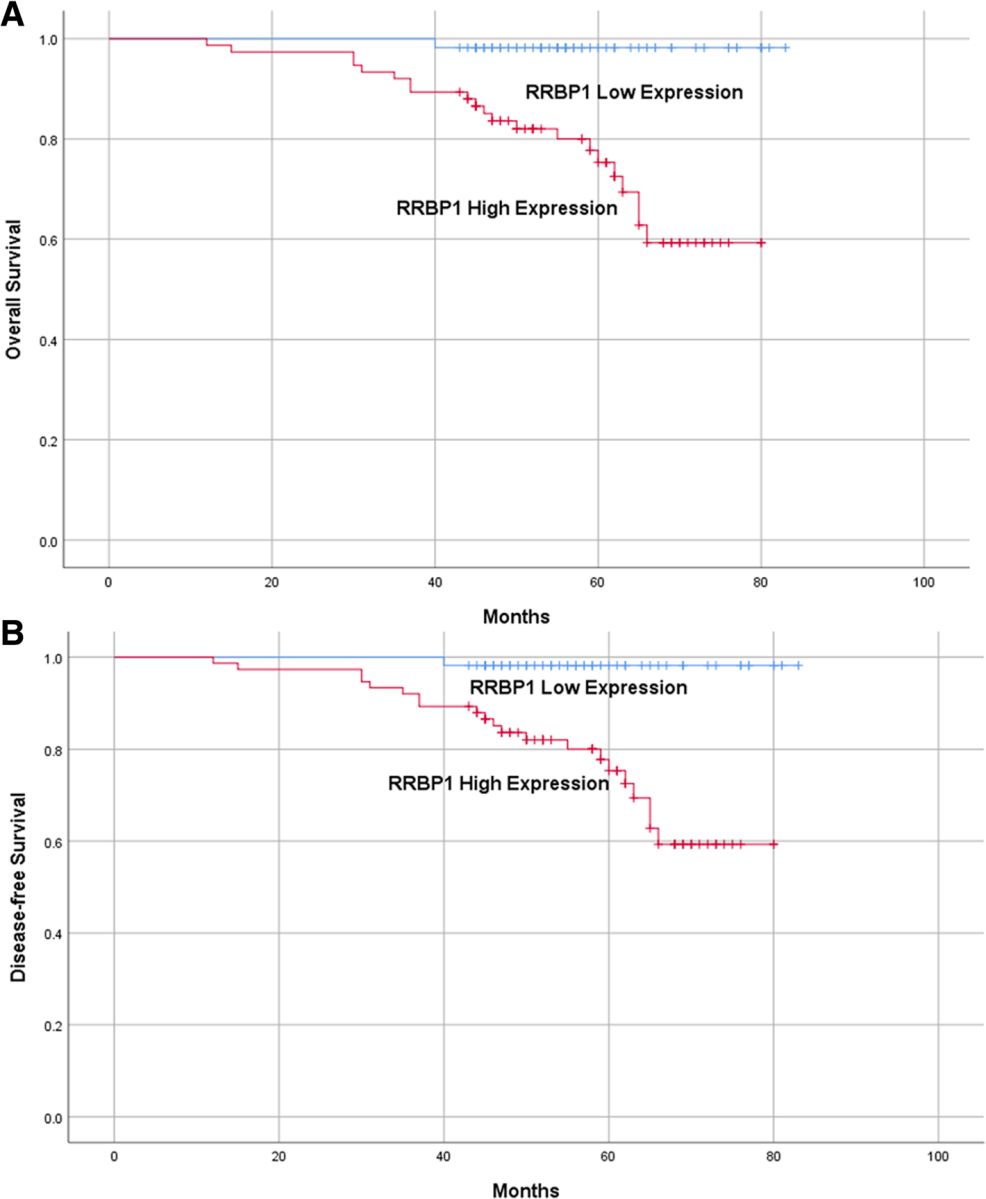
Kaplan-Meier analysis of overall survival and disease-free survival related to the expression of RRBP1. Patients with high expression of RRBP1 had a poorer prognosis than those of patients with low expression of RRBP1. **a**, overall survival curves of the EC according to their RRBP1 expression status, *P* = 0.001; **b**, disease-free survival curves of the EC patients according to their RRBP1 expression status, *P* < 0.001

Through log-rank test analysis, we found that high expression of RRBP1 had a strong correlation with poor overall survival (OS) and disease-free survival (DFS) in EC patients (Table 2; Fig. 3; *P* = 0.001 and *P* < 0.001). Through multivariate analysis, we also found that high RRBP1 expression was an independent prognostic factor for both OS and DFS (Table 3; *P* = 0.033 and *P* = 0.016).

**Table 2:** Univariate survival analysis of OS and DFS in patients with endometrial endometrioid adenovarcinoma

Variables	*n*	OS		*P* ^a^	DFS		*P* ^a^
		Mean ± SE(month)	95% CI		Mean ± SE(month)	95% CI	
Age(years)							
<60	93	77 ± 2	74–80	*P* = 0.872	74 ± 2	70–78	*P* = 0.695
≥ 60	37	75 ± 2	72–79		74 ± 2	69–78	
FIGO stage							
I	103	78 ± 1	75–80	*P* <0.001	76 ± 1	73–79	*P* <0.001
II	17	76 ± 3	70–82		72 ± 4	64–80	
III	10	52 ± 3	46–58		45 ± 5	35–54	
Histological grade							
G1	42	78 ± 2	73–82	*P* = 0.593	78 ± 2	74–83	*P* = 0.160
G2~ G3	88	75 ± 1	74–79		71 ± 2	67–75	
Lymph node metastasis							
No	122	78 ± 1	75–80	*P* <0.001	77 ± 1	74–80	*P* <0.001
Yes	8	51 ± 4	44–58		40 ± 5	30–50	
Deep muscular layer infiltration							
<50%	107	78 ± 1	76–81	*P* = 0.001	77 ± 2	74–80	*P* = 0.002
≥ 50%	23	66 ± 3	60–72		61 ± 4	53–69	
RRBP1							
Low expression	55	82 ± 1	81–84	*P* = 0.001	82 ± 1	81–84	*P* <0.001
High expression	75	72 ± 2	74–79		68 ± 2	72–78	

FIGO, International Federation of Gynecology and Obstetrics; G1, well differentiated; G2, moderately differentiated; G3, poorly differentiated; RRBP1, Ribosome-binding protein 1; OS, overall survival; DFS, disease-free survival; ^a^Log-rank test

**Table 3:** Multivariate survival analysis of OS and DFS in patients with endometrial endometrioid adenovarcinoma

Variables	OS			DFS		
	Exp(B)	95% CI	*P* ^a^	Exp(B)	95% CI	*P* ^a^
RRBP1	9.456	1.202–74.352	*P* = 0.033	12.059	1.592–91.327	*P* = 0.016
Lymph node metastasis	20.813	4.895–88.502	*P* <0.001	8.698	3.121–24.237	*P* <0.001

FIGO, International Federation of Gynecology and Obstetrics; G1, well differentiated; G2, moderately differentiated; G3, poorly differentiated; RRBP1, Ribosome-binding protein 1; OS, overall survival; DFS, disease-free survival; ^a^Cox regression test

## Discussion

As far as we known, this is the first study to investigate RRBP1 expression in endometrial carcinoma and normal endometrium tissues. We found that RRBP1 is overexpressed in EC patients, and its expression is correlated with tumor progression and poor survival.

In our current research, western blotting indicated that RRBP1 is highly expressed in EC cases and weakly expressed in normal samples. We analyzed the association between RRBP1 expression levels and a range of clinicopathologic features including FIGO stage, lymph node metastasis and depth of myometrial in endometrioid-type endometrial carcinoma (EC). In addition, patients with RRBP1 high expression had a shorter duration of OS than patients with RRBP1 low expression. Thus, RRBP1 may be a valuable biomarker for predicting EC progression and patient prognosis. Our findings are in agreement with the previous studies on the roles of RRBP1 in tumor progression in various cancers, such as lung cancer [8], breast cancer [9], colorectal cancer [10] and esophageal cancer [11].

There is growing evidence that RRBP1 plays a multifaceted role in cancer progression. There is also evidence that RRBP1 is an important ingredient that enhances tumorigenicity both in vitro and in vivo. Knockdown of RRBP1 mRNA in an orthotopic lung model significantly reduced its tumorigenicity [8]. Jen-Chieh Lee et al. reported that RRBP1-ALK and RANBP2-ALK are the only recurrent oncogene mechanisms identified in EIMS so far [15]. It has been reported that the IRES activity of 51 UTR of RRBP1 mRNA enhances the expression of RRBP1 protein, which makes hepatoma cell BEL7402 cells play a role in cellular immunity and promote the occurrence of liver cancer [16]. It has also been reported that RRBP1 may be involved in the development of acute myeloid leukemia [17].

This study also has several limitations. First of all, only a relative small sample size was available in our study. Secondly, it is a retrospective study without the mechanism research. The third disadvantage was that only patients with endometrioid-type endometrial endometrioid adenovarcinoma were included in our study. Therefore, a much larger study would needed to effectively test our conclusion, and most importantly, investigate the RRBP1 expression in any of the other histologic subtypes.

In summary, this research suggested that overexpression of RRBP1 is closely correlated with a poor prognosis of EC patients. RRBP1 may become a useful target for treating endometrial cancer and a marker for identifying patients with poor prognoses. This conclusion needs additional experiments conducted to develop a better test for the biomarker and to validate the results.

## Conclusion

This experiment identifies the utility of RRBP1 in predicting EC prognosis, revealing that it may be a potential target for therapeutics of EC.
